# Potential inflammatory mechanisms of the ketogenic diet against febrile infection-related epilepsy syndrome

**DOI:** 10.1186/s42494-024-00187-y

**Published:** 2025-01-10

**Authors:** Juan Wang, Lingling Xie, Li Jiang

**Affiliations:** 1 Department of Neurology, Children’s Hospital of Chongqing Medical University, National Clinical Research Center for Child Health and Disorders, Ministry of Education Key Laboratory of Child Development and Disorders, No. 136, Zhongshan 2nd Road, Yuzhong District, Chongqing, 400014 China; 2 Chongqing Key Laboratory of Neurodevelopment and Cognitive Disorders, Chongqing, 400014 China

**Keywords:** Ketogenic diet, Febrile infection-related epilepsy syndrome, Inflammation, Mechanisms

## Abstract

Febrile infection-related epilepsy syndrome (FIRES) is a rare epilepsy syndrome with unclear pathogenesis, characterized by fever-induced, super-refractory status epilepticus and high mortality. Studies have shown that ketogenic diet (KD) is effective in controlling convulsions in FIRES, but its mechanisms are unclear. This paper intends to summarize the mechanisms by which KD may exert effects against FIRES. Clinical studies have shown that patients with FIRES have elevated levels of various inflammatory factors such as interleukin (IL)-6, IL-8, IL-10, and so on. KD may exert anti-FIRES effects through several potential inflammatory pathways, including nuclear factor -κB (NF-κB) and NLR family pyrin domain containing 3 (NLRP3). Furthermore, the Kyoto Encyclopedia of Genes and Genomes (KEGG) network suggested that KD may play an anti-inflammatory role through several pathways such as cellular senescence and neutrophil extracellular trap formation. These mechanisms need to be further investigated.

## Background

Febrile infection-related epilepsy syndrome (FIRES) is categorized as a subtype of new onset refractory status epilepticus (NORSE) according to the definition of the International League Against Epilepsy (ILAE) [1]. Its mortality rate is as high as 10–30% [2, 3]. FIRES is a rare disorder characterized by unknown etiology, lack of specific biomarkers, and widespread drug resistance [2]. The ketogenic diet (KD) is a specialized dietary regimen designed to maintain a continuous production of ketone bodies in the body through a ratio of high fat, moderate protein and low carbohydrate [4]. This diet has been widely used in the treatment of refractory epilepsy, especially in pediatric patients with significant efficacy [5]. Although KD has multiple biological pathways of action [6], its specific mechanism of antiepileptic activity varies depending on the etiology of the disease. The FIRES Expert Consensus [7] and the KD Guidelines [8] recognize the effectiveness of KD in the treatment of FIRES and recommend it as a therapeutic tool. However, the specific mechanisms by which KD acts in the treatment of FIRES remain unknown. Several clinical studies have shown that KD is effective in treating FIRES and that the levels of several inflammatory factors, such as interleukin (IL)-6, IL-8, and IL-10, are elevated in patients with FIRES [9–12]. Meanwhile, KD may exert its anti-inflammatory effects through multiple pathways [13–16]. These results suggest that inflammation may be the potential mechanisms of action for KD against FIRES. An in-depth investigation into the mechanisms of KD in treating FIRES is of critical importance to reduce the risk of mortality and may provide an important scientific basis for the clinical treatment of FIRES.

This paper aims to review evidence of inflammation in FIRES and the anti-inflammatory mechanisms of KD to access the possible mechanisms of KD may treat FIRES.

## Inflammatory evidence in FIRES

### Clinical studies

Clinical studies have shown a significant correlation between systemic inflammatory states and persistent convulsive states in patients in adult intensive care units [17]. Inflammation is not only a potential cause of epilepsy, but also indicators of inflammation may be associated with a variety of brain disorders, including epilepsy [18, 19]. In addition, neuroinflammatory pathways have been recognized as potential targets for epilepsy treatment [20], and anti-inflammatory therapy can enhance the efficacy of antiseizure medications [21]. A study of patients with NORSE found elevated levels of cytokines (e.g., IL-6, tumor necrosis factor alpha [TNF-α], IL-8, C–C motif ligand 2 [CCL2], and macrophage inflammatory protein 1 alpha [MIP-1α]) [22].

FIRES, a subtype of new onset refractory status epilepticus (NORSE) [1], exhibits a range of clinical features associated with inflammation, although the etiology is not yet clear. Patients usually have a documented fever 24 h to 2 weeks prior to an attack, may present with a persistent convulsive state that is widely resistant to conventional antiseizure medications and sedatives, and may even show resistance to anesthetic drugs. At the same time, some patients are effectively treated with immunosuppressive agents. The University of Cincinnati College of Medicine reported a patient with ultra-refractory persistent status epilepticus who experienced almost complete relief of seizures after treatment with the IL-1 receptor antagonist and the IL-1β monoclonal antibody [23]. A case report from Harvard Medical School also found that IL-6 blockers were effective in the treatment of FIRES [24]. Based on evidence from several studies, including the 2022 FIRES Expert Consensus Recommendations [7], IL-1 receptor antagonists and IL-6 blockers have been included as effective second-line treatments for FIRES. These findings suggest that there may be a correlation between FIRES and inflammatory response.

Given the low prevalence of FIRES, current research is dominated by case reports or small sample studies. Although these studies have shown that patients with FIRES may have elevated levels of a variety of inflammatory factors, the elevation of these inflammatory factors varies between studies. For example, in a study of an 18-year-old male patient with FIRES, his cerebrospinal fluid and serum cytokine assays showed significant elevations of inflammatory factors such as IL-6, IL-1, monocyte chemotactic protein-1 (MCP-1), macrophage inflammatory protein 1β (MIP-1β), and interferon gamma (IFN-γ), primarily in the central nervous system [9]. In another study involving six patients with FIRES, the levels of inflammatory factors such as Th1-related cytokines/chemokines (e.g., TNF-α, IL-9, IL-10, IL-11, IL-6, CCL2, CCL19, and IL-1) were significantly elevated [10]. In addition, a study of six patients with FIRES in the acute phase and one patient with FIRES in the chronic phase also noted significantly elevated levels of inflammatory factors such as IL-6, IL-8, IL-1β, IL-10, IL-9, and IFN-γ [11]. In a patient from Minnesota, serum tests showed elevated levels of inflammatory factors such as IL-6, IL-8, high mobility group protein 1 (HMGB1), and S100 calcium-binding protein A8/A9 (S100A8/A9), while elevated levels of IL-6 were detected in his cerebrospinal fluid [12]. In pediatric patients with FIRES, peripheral blood mononuclear cells and monocyte-derived dendritic cells exhibit impaired Toll-like receptor (TLR) responses, including responses to TLR3, TLR4, TLR7/8, and TLR9 [25].

### Laboratory studies

Due to the lack of FIRES-specific animal models, there is a scarcity of laboratory research on FIRES, and no FIRES-related inflammatory pathways have been reported for the time being. As one of the epilepsy syndromes, FIRES is likely to share potential inflammatory pathways with epilepsy. Therefore, we reviewed the inflammatory mechanisms of epilepsy.

#### Epilepsy upregulates inflammatory signaling pathways

Seizures are closely linked to the activation of multiple inflammatory pathways. It has been shown that inhibition of these associated pathways effectively reduces seizures, which has been consistently confirmed in multiple experimental epilepsy models [26]. Two studies using rat models of amygdala-ignited epilepsy found that inflammation was upregulated during epileptogenesis primarily through the NLR family pyrin domain containing (NLRP)1, NLRP3, caspase 1 and IL-1β pathways [27, 28]. Two studies in the pentetrazol model confirmed that inflammation was upregulated mainly through the NLRP3, caspase 1, apoptosis-associated speck-like protein with a caspase-recruitment domain (ASC) and IL-1β pathways [29, 30]. In the kainic acid epilepsy mouse model, the results of the three studies collectively point to the signaling pathways of NLRP1, NLRP3, absent in melanoma 2 (AIM2), caspase 1, and ASC playing a key role in the activation of inflammation during epilepsy [31–33]. In a mouse model of pilocarpine-induced epilepsy, four studies consistently show that inflammation is enhanced through NLRP3, caspase 1, ASC, gasdermin D, IL-1β, and IL-18 pathways [34–37]. Brain tissue studies in patients with temporal lobe epilepsy (TLE) have also found that signaling pathways such as NLRP1, NLRP3, caspase 1, ASC and IL-1β play an important role in the enhancement of inflammation [37, 38].

#### Inflammasomes deteriorate epilepsy

The important role of inflammasomes as characteristic markers of epileptic susceptibility has been demonstrated in several experimental models. For example, in the rodent model of TLE induced by amygdala ignition [27], the epilepsy model applying the excitatory neurotransmitter agonists kainic acid [33] and pilocarpine [36], and the model involving inhibition of inhibitory neurotransmitters using pentylenetetrazole [29]. Furthermore, it has been found that activation of the NLRP1, NLRP3, or AIM2 inflammasomes leads to the worsening of epileptic seizures [31, 32, 34]. In particular, seizures were effectively controlled by decreasing the levels of AIM2 and NLRP3 and NLRP1 in a mouse model of kainic acid-induced epilepsy [32]. In addition, indirect targeting therapy against NLRP3 has obtained similar results in rodent models. In a mouse model of pilocarpine-induced epilepsy, activation of the transcriptional repressor Rev-erbalpha decreased NLRP3 expression in the brain, reduced NLRP3-mediated inflammatory vesicle activation, and inhibited astrocyte proliferation and neuronal death [37]. Similarly, in this model, inhibition of NLRP3 using MCC950 reduces levels of NLRP3, ASC and caspase 1 in the brain [35]. In the amygdala-ignited rat epilepsy model, knockdown of *NLRP1*, *NLRP3* and *Caspase 1* genes by using siRNA technology also reduced epileptic activity [27, 28]. The deubiquitinase USP47, which specifically inhibits NLRP3 activation, was also able to inhibit NLRP3 activation, thereby ameliorating seizures in rats [36].

#### Calcium ion

A large body of research evidence suggests that the activation of NLRP3 in epilepsy models is closely related to the inward flow of calcium ion. A study in a mouse model of cerebral hemorrhage found that the activation of transient receptor potential channel subfamily V member 4 (TRPV4) channels increased intracellular calcium ion concentration and further induced the release of endoplasmic reticulum stress markers [39]. In addition, a mouse model induced by pilocarpine verified the correlation between elevated intracerebral calcium ion levels and increased levels of inflammasomes components [37]. In the same model, blockade of TRPV4 channels using HC-067047 was effective in inhibiting calcium ion inward flow and attenuating inflammatory vesicle activation [34]. On the other hand, in a mouse model of kainic acid-induced epilepsy, treatment with JNJ-47965567 to inhibit the calcium-sodium ion channel P2X7 receptor has also been shown to attenuate seizures [40].

#### sTNFr2

Soluble tumor necrosis factor receptor 2 (sTNFr2) is one of the soluble members of the TNFα receptor family, and is regarded as a potent circulating marker reflecting TNFα activity due to its high stability [41]. TNFα is a peptide cytokine secreted by monocytes, macrophages and T lymphocytes, and is primarily responsible for immunomodulation [42]. sTNFr2 is an important biomarker for assessing TNFα activity. Clinical studies have demonstrated that plasma levels of sTNFr2 correlate with seizure frequency and are effective in differentiating epileptic from non-epileptic patients [19].

However, it is noteworthy that the upregulation of inflammatory markers observed in rodent models of epilepsy is not consistent with findings in patients with TLE. Studies in mice have revealed that diet plays a key role in modulating inflammasome activation in epilepsy models. In particular, in PTZ-induced or kainic acid-induced mouse models of epilepsy, the addition of the omega-3 fatty acids (such as docosapentaenoic acid and docosahexaenoic acid) to the diet was able to inhibit the binding of ASCs to NLRP3 and reduce the production of IL-1β, which effectively protects the mice from the effects of epilepsy [29, 33]. Numerous studies have also found that the high activation of NLRP3 and NLRP1 inflammatory vesicles in the brain and blood of epileptic patients leads to significantly elevated levels of ASC, caspase 1 and IL-1β [43–45]. Additionally, it has been shown that lesion-associated epilepsy causes further activation of inflammasomes through the HMGB1/TLR4 and IL-1β/IL-1R1 signaling pathways [45]. However, when brain tissue from TLE patients was studied, it was found that there were no significantly elevated levels of NLRP3, caspase 1 or IL-1β [31].

## Anti-inflammatory mechanisms of KD

### Inhibition of the NLRP3 signaling pathway

It has been hypothesized that microglia are closely associated with sterile neuroinflammation and that hyperactivation of the NLRP3 inflammasome/IL-1 axis in microglia generates a pro-inflammatory and pro-convulsant endo-environment, which contributes to the initiation of FIRES [46]. In a study of human focal epilepsy, investigators found an increased number of NLRP3-expressing CD3^+^ and CD14^+^ cells in peripheral blood mononuclear cells from epilepsy patients [47]. Given that FIRES is a special type of epilepsy characterized by multifocal focal seizures with unknown etiology, in addition to the inflammatory features described above, it can be determined that NLRP3 inflammasome may be associated with the development of FIRES. And previous studies have found that KD treatment has an anti-NLRP3 inflammasome effect [13]. Whether KD exerts an anti-FIRES effect by acting on NLRP3 inflammasome needs to be further investigated.

Activation of NLRP3 inflammatory vesicles can increase the release of pro-inflammatory cytokines. In a clinical trial in healthy subjects, it was found that 3-day short-term KD significantly reduced interleukin 1 beta (IL-1β) and TNF-α secretion induced by adenosine triphosphate or palmitate stimulation in human macrophages, and that beta-hydroxybutyric acid (BHB), the main product of KD, could exert anti-inflammatory effects by inhibiting the activation of the NLRP3 inflammatory vesicle and its associated signaling pathway [13]. In addition, in an in vitro model of glioma cells, BHB was shown to reduce the levels of activated cysteine aspartate-specific protease 1 and mature IL-1β, which in turn inhibited the migration of C6 glioma cells in vitro and the activation of NLRP3 inflammasomes, thereby reducing the inflammatory microenvironment and the inflammatory response [14]. Aside from direct effects on inflammatory vesicles, studies of adipose tissue-resident immune cells in mice revealed that KD expanded metabolically protective γδ T cells with anti-inflammatory properties, further exerting anti-inflammatory effects [15]. In a rat model of ulcerative colitis, KD was found to exert antioxidant effects by reducing reactive oxygen species production and improving the activity of antioxidant enzymes [16]. From mRNA and protein expression analyses, the KD therapy was found to exert anti-inflammatory effects by inhibiting the activation of NLRP3 inflammatory vesicles, reducing NLRP3/NGSDMD-mediated cellular pyroptosis, and lowering the levels of inflammatory markers, including myeloperoxidase, nuclear factor-κB (NF-κB), IL-6, and TNF-α [16].

### Anti-NF-κB signaling pathway

Abnormal formation of neural circuits and altered neuroplasticity are the main causes of epilepsy [48, 49], and NF-κB may play a key role in the inflammatory response of the nervous system [6]. The NF-κB signaling pathway, a typical pro-inflammatory response pathway, is involved in the regulation of altered plasticity of neural circuits through various mechanisms, including but not limited to neuronal survival and axon growth [50], synaptic plasticity [51], and myelin formation [52].　In a study of two types of human epilepsy, investigators found an increased number of NF-κB-expressing CD14^+^ peripheral blood mononuclear cells in patients of focal epilepsy, NF-κB mRNA expression levels were elevated, and serum levels of IL-1β and IL-6 were also increased in patients of unknown etiology [47].

Clinical studies suggest that KD may exert anti-inflammatory effects by inhibiting NF-κB expression [53, 54]. A case report from Harvard Medical School described a patient who did not respond to KD and IL-1 receptor antagonist therapy, but subsequently achieved significant results with IL-6 blockers [24]. The efficacy of IL-6 blocker therapy has also been demonstrated in two patients (one pediatric and one adult) in France [55] and in a case report of an 18-year-old patient with FIRES in Singapore [9], all of whom received KD therapy. Whether KD therapy exerts an anti-inflammatory effect through the IL-6-NF-κB signaling pathway needs to be further investigated in the future.

In a study comparing TLE with focal epilepsy of unknown cause, the IL-6-NF-κB signaling pathway was found to be activated in patients with TLE [47]. The features of focal epilepsy and status epilepticus also coincides with those of patients with FIRES. Furthermore, despite the lack of consistency in the various cytokines detected in FIRES patients, they are all downstream products of the NF-κB signaling pathway [9–12, 25]. Thus, the NF-κB pathway may be a potential mechanism of action of KD in the fight against FIRES.

KD may exert anti-inflammatory effects by inhibiting NF-κB activation. In a rat inflammation model, the KD therapy was found to directly inhibit the activation of NF-κB to attenuate the inflammatory response, as well as further attenuate the inflammatory response by decreasing the level of TNF-α in the hippocampus region and decreasing the translocation of NF-κB to the nucleus [6]. In addition, BHB produced by KD was found to activate peroxisome proliferator-activated receptor γ and inhibit the cyclooxygenase-2-dependent pathway in a mice model of alginate-induced epilepsy, thereby suppressing the neuroinflammatory response [56]. Meanwhile, in a rat model of spinal cord injury, KD was also found to inhibit the NF-κB signaling pathway through activation of Nrf2, further attenuating the inflammatory response [57]. In an obese rat model, KD was able to improve the abnormal metabolism induced by NF-κB overexpression in the hippocampus, including the metabolic imbalance caused by citrate synthase overactivation and ATP synthase downregulation [58].

Bilirubin, a potent endogenous anti-inflammatory antioxidant, has significant anti-inflammatory effects [59]. Bilirubin can freely cross the blood–brain barrier and shares the NF-κB signaling pathway with dopamine [60], exerting neuroprotective effects by lowering TNF-α levels [61]. The relationship among FIRES, KD, and bilirubin need further investigation.

In a mouse model of multiple sclerosis with metaplastic encephalomyelitis, the KD was found to attenuate neuroinflammation and promote the conversion of M1-type microglia to M2-type by modulating the NF-κB/NLRP3 pathway and inhibiting the activation of histone deacetylase 3 and P2X7 receptor [62]. Furthermore, these findings are consistent with studies using engineered nano-erythrocytes to modulate microglia polarization as an anti-inflammatory target in the central nervous system. Engineered nano-erythrocytes were able to promote the transition of microglia from M1 to M2 type, which in turn inhibited the translocation of NF-κB p65 and exerted anti-inflammatory effects. This technique has been demonstrated in models of middle cerebral artery occlusion and experimental autoimmune encephalomyelitis [63].

### Activation of the GRP109A signaling pathway

G protein-coupled receptor 109A (GPR109A) is a receptor that exhibits a wide range of expression patterns in many types of immune cells and plays an important role in the anti-inflammatory process of various diseases [64]. KD may exert anti-inflammatory effects by activating the GPR109A signaling pathway. Studies on microglia energy metabolism have shown that KD inhibits histone deacetylase activity in the central nervous system, activates microglia GRP109A receptors, inhibits microglia overactivation, and thus promotes a neuroprotective microglia phenotype [65]. However, studies on mouse models of inflammation have shown that BHB blocks NLRP3 inflammasome-mediated inflammatory disease, but this effect is not directly linked to GPR109A function [66]. Therefore, the relevance of KD to the GRP109A signaling pathway needs to be confirmed by further studies.

### Inhibition of cytokine storm pathways

In studies targeting coronavirus disease 2019, researchers have observed that KD has the ability to inhibit and even prevent cytokine storms [67, 68]. In addition, in a mouse model of inflammation, KD was found to mitigate cytokine storms by promoting protective γδ T-cell responses as well as increasing the expression of electron transport chain genes [69]. Gout-related studies have also found that KD effectively reduces the levels of pro-inflammatory cytokines such as IL-1β and TNF-α, exerting an anti-inflammatory effect [70]. This mechanism of action has also been demonstrated in obese female patients, where KD significantly reduced IL-1β levels [71].

### Regulation of macrophage transformation pathways

Studies of microglia energy metabolism have shown that KD therapy decreases glucose metabolism, which in turn reduces the macrophage NADH/NAD^+^ ratio, NF-κB transcriptional activity, and pro-inflammatory gene expression [65]. In a mouse model of glioblastoma, KD was found to increase immunosuppressive M2 macrophages by 50% and decrease pro-inflammatory M1 macrophages by 50% [72]. Another research in the mouse model of glioblastoma from the same institution showed similar findings, which were consistent with the in vitro and in vivo results, demonstrating a paradoxical 50% increase in immunosuppressive M2 macrophages (CD45^+^CD11b^+^F4/80^+^CD206^+^) and a concomitant decrease in pro-inflammatory M1 macrophages (CD45^+^CD11b^+^F4/80^+^CD80^hi^) due to KD [73]. In addition, in a stroke model, ketone bodies were found to activate a neuroprotective phenotype in macrophages by activating the macrophage ketone receptor hydroxycarboxylic acid receptor 2, which promotes cyclooxygenase 1 and hematopoietic prostaglandin D2 (PGD2) synthase-dependent production of PGD2 [74]. In a prostate cancer model, ketone bodies were also shown to act as endogenous histone deacetylases (HDACs) inhibitors, significantly reducing M2-polarized immunosuppressive macrophages, thereby exerting an anti-inflammatory effect [75].

### Inhibition of HDACs signaling pathway

HDACs are enzymes that regulate chromatin structure and accessibility. In models of Alzheimer's disease, ketone bodies produced by KD have been found to inhibit the activity of HDACs, exerting anti-inflammatory effects [76]. Studies on mammalian stem cell models have shown that KD enhances Notch signaling pathway mainly by inhibiting HDAC, which in turn promotes cell self-renewal, function and regeneration after injury [77]. In a model of middle cerebral artery occlusion and experimental autoimmune encephalomyelitis, microglia are an important target for anti-inflammatory therapy [63]. In a model of neuroinflammation induced by lipopolysaccharides or chronic unpredictable stress, the inhibitory effect of KD on HDAC was found to activate the protein kinase B-microGTPase Rho axis, triggering changes in microglia and thus producing an anti-inflammatory effect [78]. Additionally, KD, as an endogenous HDAC1 inhibitor, was found to increase histone acetylation levels in neurons and improve *HDAC2* gene overexpression in a mouse model of autism [79]. Studies in a rat model of spinal cord injury found that KD attenuated oxidative stress through inhibition of HDAC1 and modulated the expression of FOXO3a, NOX2 and NOX4 through selective inhibition of HDAC1 or HDAC2 [80].

### Regulated gene pathways

Studies of oxidative stress in mice have shown that KD increases histone acetylation levels of genes encoding antioxidant stress response factors, thereby exerting their anti-inflammatory effects [81]. In addition, studies in adult rat models of inflammation have shown that KD regulates the innate inflammatory response through the transcriptional co-blocker CtBP: during KD, glucose metabolism is reduced, leading to a decrease in the NADH/NAD^+^ ratio in macrophages and microglia, which in turn reduces the transcriptional activity of NF-κB and the expression of pro-inflammatory genes. The change in the NADH/NAD^+^ ratio affects the binding of CtBP to the acetyltransferase p300, which in turn regulates the acetylation level of the NF-κB p65 subunit, thereby affecting its binding to the promoters of pro-inflammatory genes [82]. Genetic testing of 19 FIRES patients in Japan showed that *IL1RN* rs4251981 G > A and *SCN2A* rs1864885 A > G may be potentially associated with FIRES [83]. It is well known that KD modulates sodium channels [84]. Whether KD exerts therapeutic effects on FIRES by modulating sodium channels needs to be confirmed by more studies.

### Regulation of mitochondrial pathways

Studies in spontaneously epileptic *Kcna1*-deficient mice have shown that BHB regulates mitochondrial permeability transition [85]. Besides, in a rat model of irritable bowel syndrome, KD was also found to alleviate the inflammatory response and improve intracellular redox homeostasis by elevating the activity of the peroxisome proliferator-activated receptors-γ/proliferator-activated receptor gamma co-activator-1 alpha signaling pathway, which restored the normal function of mitochondria [86]. KD also reduced the degree of mitochondrial DNA methylation. The results of RNAseq analysis indicated that this effect may originate from the regulatory effect of KD on mitochondrial translation and electron transport chain-related pathways [87].

### Regulation of intestinal flora pathways

Studies in a mouse model of dextran sodium sulfate colitis have confirmed that KD exerts anti-inflammatory effects by influencing the gut microbiota to reduce inflammatory cytokines (e.g., IL-17α, IL-18, IL-22, and CCL-4) produced by innate lymphoid cells of the RORγt^+^CD3^−^ group 3 [88]. What’s more, KD can also exert anti-inflammatory effects by modulating a variety of anti-inflammatory pathways produced by gut flora, including but not limited to the FGF21-β-hydroxybutyrate-NLRP3 axis, the GCN2-eIF2α-ATF4 pathway, the von Hippel-Lindau/Hypoxia-inducible transcription factor pathway, and the TMAO-PERK -FoxO1 axis [89].

**Fig. 1:**
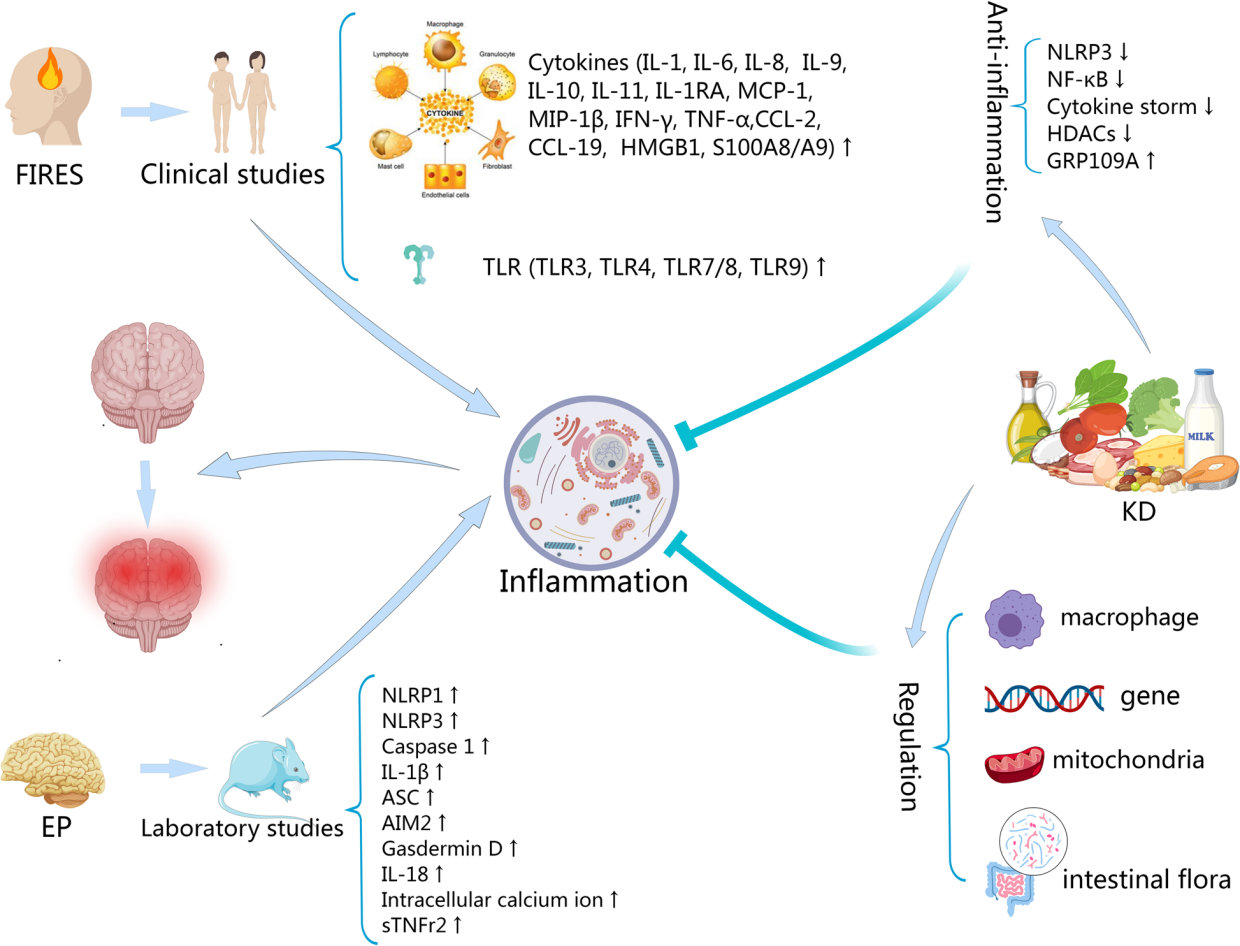
Potential inflammatory pathways for KD against FIRES. Clinical studies on FIRES have shown that the levels of cytokines and Toll-like receptors are elevated. Laboratory studies on epilepsy have shown that the levels of inflammasomes are also elevated. Meanwhile, KD may exert its anti-inflammatory effects through multiple pathways. AIM2 Absent in melanoma 2. ASC Apoptosis-associated speck-like protein with a caspase-recruitment domain. CCL C–C motif ligand. EP Epilepsy. FIRES Febrile infection-related epilepsy syndrome. GPR109A G protein-coupled receptor 109A. HDACs Histone deacetylases. HMGB1 High Mobility Group Protein 1. IFN-γ Interferon gamma. IL Interleukin. KD Ketogeic diet. MCP Monocyte chemotactic protein. MIP Macrophage inflammatory protein. NF-κB Nuclear factor-κB. NLRP NLR family pyrin domain containing. S100A8/A9 S100 calcium-binding protein A8/A9. sTNFr2 Soluble tumor necrosis factor receptor 2. TLR Toll-like receptor. TNF-α Tumor necrosis factor alpha

According to the current reports, clinical studies on FIRES have shown that the levels of cytokines (IL-1, IL-6, IL-8, IL-9, IL-10, IL-11, IL-1RA, MCP-1, MIP-1β, IFN-γ, TNF-α, CCL-2, CCL-19, HMGB1, S100A8/A9) and Toll-like receptors (TLR3, TLR4, TLR7/8, and TLR9) were elevated. Laboratory studies from epilepsy have shown that the levels of inflammasomes (NLRP1, NLRP3, caspase 1, IL-1β, ASC, AIM2, gasdermin D, IL-18, intracellular calcium ion, sTNFr2) were also elevated. Meanwhile, KD may exert its anti-inflammatory effects through multiple pathways including the inhibition of NLRP3, NF-κB, cytokine storm, and HDACs; activation of GRP109A; and regulation of macrophage transformation, gene expression, mitochondria function, and intestinal flora (see Fig. 1).

#### Other potential pathways

**Fig. 2:**
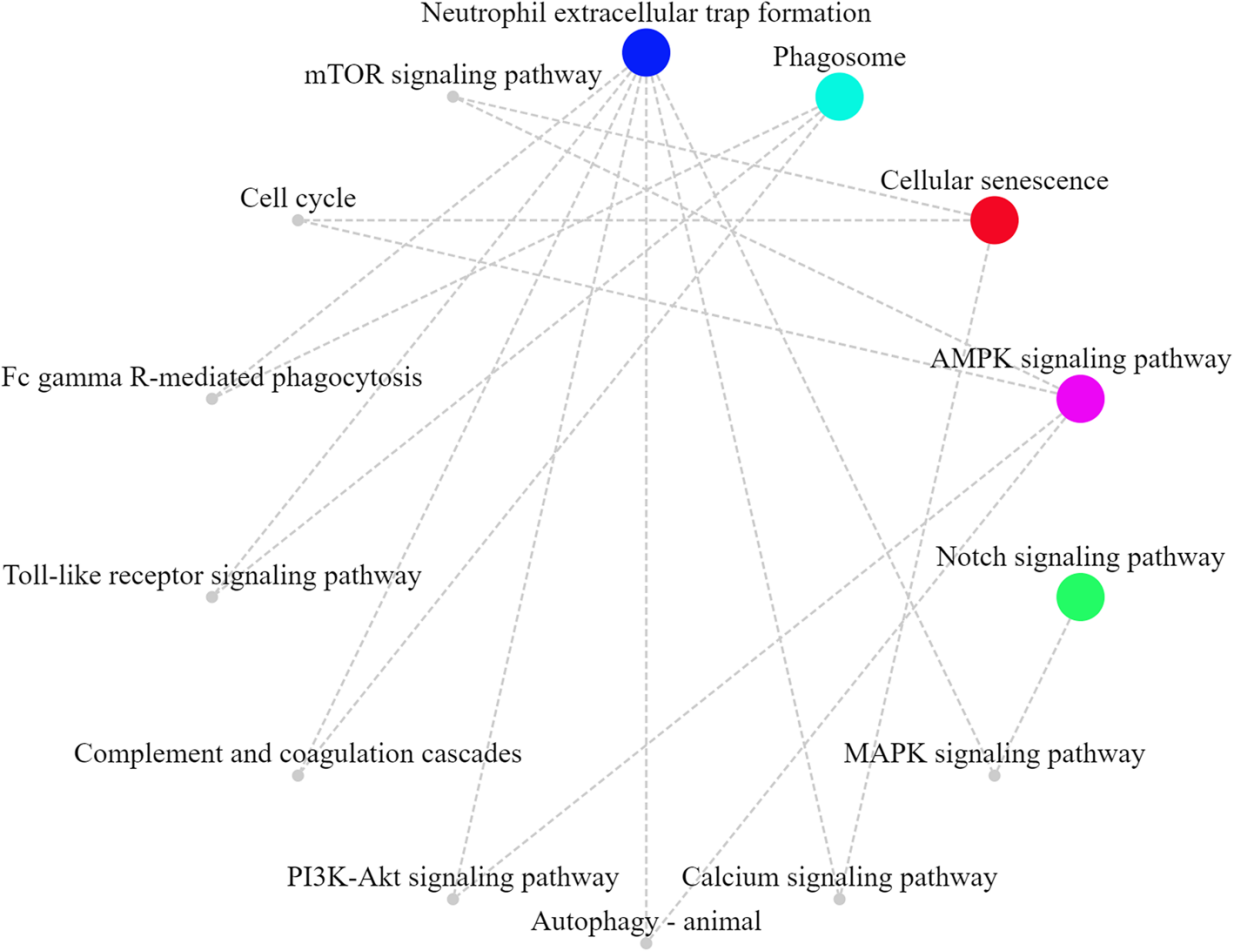
KEGG network for possible mechanisms of KD against FIRES. KD may play its anti-inflammatory role through pathways, either directly or indirectly. AMPK Adenosine monophosphate-activated protein kinase. MAPK Microtubule-associated protein kinase

In addition to the existing reports mentioned above, there may be unreported potential anti-inflammatory mechanisms of KD that contribute to its effects against FIRES. Based on the concepts of "central immunity", "central inflammation", and "KD anti-inflammatory mechanisms", we searched the signaling pathways in the Kyoto Encyclopedia of Genes and Genomes (KEGG) signaling database (the KEGG Orthology numbers were: map04218, map05417, map05417, map03320, map04152, map07051, map05417, map04330, map04613, map04145, and map05418, respectively). We further used OmicShare Tools (https://www.omicshare.com/tools) to draw a KEGG network to observe the general situation of the signaling pathways. As shown in the Fig. 2, KD may play an anti-inflammatory role through a variety of signaling pathways, including cellular senescence, neutrophil extracellular trap formation, phagosome, adenosine monophosphate-activated protein kinase (AMPK), Notch, microtubule-associated protein kinase (MAPK), calcium, mTOR, cell cycle, Fc gamma R-mediated phagocytosis, Toll-like receptors, complement and coagulation cascades, PI3K-Akt, autophagy- animal, either directly or indirectly.

## Outlook

Despite the overview of the possible underlying mechanisms of KD against FIRES, these mechanisms need further validation. Currently, due to the lack of animal models of FIRES, studies on the relevant mechanisms must be conducted using epilepsy models. Considering that FIRES is only one of many rare epilepsy syndromes, whether the findings from epilepsy models are fully applicable to FIRES patients still needs to be confirmed by in-depth mechanism studies. In addition, the consistency between laboratory research and clinical practice needs to be further explored.

## Conclusions

In conclusion, clinical studies have shown that patients with FIRES have elevated levels of various inflammatory factors such as IL-6, IL-8, and IL-10. KD may exert anti-FIRES effects through several potential inflammatory pathways, such as NF-κB and NLRP3. Furthermore, KEGG network analysis showed that KD may play an anti-inflammatory role through several pathways, including cellular senescence, neutrophil extracellular trap formation, phagosome, AMPK, Notch, MAPK, calcium, mTOR, cell cycle, Fc gamma R-mediated phagocytosis, Toll-like receptors, complement and coagulation cascades, PI3K-Akt, autophagy- animal. However, these mechanisms need further investigation.

## Data Availability

Not applicable.

## Abbreviations

AIM2: Absent in melanoma 2
AMPK: Adenosine monophosphate-activated protein kinase
ASC: Apoptosis-associated speck-like protein with a caspase-recruitment domain
BHB: Beta-Hydroxybutyric acid
CCL: C-C motif ligand
FIRES: Febrile infection-related epilepsy syndrome
GPR109A: G protein-coupled receptor 109A
HDACs: Histone deacetylases
HMGB1: High Mobility Group Protein 1
IFN-γ: Interferon gamma
IL1RA: Interleukin-I receptor antagonist
IL-1β: Interleukin 1 beta
IL-6: Interleukin-6
ILAE: International League Against Epilepsy
KD: Ketogenic diet
KEGG: Kyoto Encyclopedia of Genes and Genomes
MAPK: Microtubule-associated protein kinase
MCP: Monocyte chemotactic protein
MIP: Macrophage inflammatory protein
NF-κB: Nuclear factor-κB
NLRP3: NLR family pyrin domain containing 3
NORSE: New onset refractory status epilepticus
PGD2: Prostaglandin D2
S100A8/A9: S100 calcium-binding protein A8/A9
sTNFr2: Soluble tumor necrosis factor receptor 2
TLE: Temporal lobe epilepsy
TLR: Toll-like receptor
TNF-α: Tumor necrosis factor alpha
TRPV4: Transient receptor potential channel subfamily V member 4

## References

[1] Specchio N, Wirrell EC, Scheffer IE, Nabbout R, Riney K, Samia P, et al. International League Against Epilepsy classification and definition of epilepsy syndromes with onset in childhood: Position paper by the ILAE Task Force on Nosology and Definitions. Epilepsia. 2022;63(6):1398–442. 35503717 10.1111/epi.17241

[2] Wickstrom R, Taraschenko O, Dilena R, Payne ET, Specchio N, Nabbout R, et al. International consensus recommendations for management of new onset refractory status epilepticus ( NORSE) including febrile infection-related epilepsy syndrome (FIRES): summary and clinical tools. Epilepsia. 2022;63(11):2827–39. 35951466 10.1111/epi.17391 PMC9826478

[3] Fetta A, Crotti E, Campostrini E, Bergonzini L, Cesaroni CA, Conti F, et al. Cannabidiol in the acute phase of febrile infection-related epilepsy syndrome (FIRES). Epilepsia Open. 2023;8(2):685–91. 37042946 10.1002/epi4.12740 PMC10235155

[4] Li Z, Heber D. Ketogenic diets. JAMA. 2020;323(4):386. 31990316 10.1001/jama.2019.18408

[5] Lee MB, Hill CM, Bitto A, Kaeberlein M. Antiaging diets: separating fact from fiction. Science. 2021;374(6570):eabe7365. 34793210 10.1126/science.abe7365 PMC8841109

[6] Zhu H, Bi D, Zhang Y, Ong C, Du J, Wu X, et al. Ketogenic diet for human diseases: the underlying mechanisms and potential for clinical implementations. Signal Transduct Target Ther. 2022;7(1):11. 35034957 10.1038/s41392-021-00831-w PMC8761750

[7] Sculier C, Barcia Aguilar C, Gaspard N, Gaínza-Lein M, Sánchez Fernández I, Amengual-Gual M, et al. Clinical presentation of new onset refractory status epilepticus in children (the pSERG cohort). Epilepsia. 2021;62(7):1629–42. 34091885 10.1111/epi.16950 PMC8362203

[8] Kossoff EH, Zupec-Kania BA, Auvin S, Ballaban-Gil KR, Christina Bergqvist AG, Blackford R, et al. Optimal clinical management of children receiving dietary therapies for epilepsy: Updated recommendations of the International Ketogenic Diet Study Group. Epilepsia Open. 2018;3(2):175–92. 29881797 10.1002/epi4.12225 PMC5983110

[9] Goh Y, Tay SH, Yeo LLL, Rathakrishnan R. Bridging the gap: tailoring an approach to treatment in febrile infection-related epilepsy syndrome. Neurology. 2023;100(24):1151–5. 36797068 10.1212/WNL.0000000000207068 PMC10264048

[10] Kothur K, Bandodkar S, Wienholt L, Chu S, Pope A, Gill D, et al. Etiology is the key determinant of neuroinflammation in epilepsy: elevation of cerebrospinal fluid cytokines and chemokines in febrile infection-related epilepsy syndrome and febrile status epilepticus. Epilepsia. 2019;60(8):1678–88. 31283843 10.1111/epi.16275

[11] Horino A, Kuki I, Inoue T, Nukui M, Okazaki S, Kawawaki H, et al. Intrathecal dexamethasone therapy for febrile infection-related epilepsy syndrome. Ann Clin Transl Neurol. 2021;8(3):645–55. 33547757 10.1002/acn3.51308 PMC7951105

[12] Howe CL, Johnson RK, Overlee BL, Sagen JA, Mehta N, Farias-Moeller R. Drug-resistant seizures associated with hyperinflammatory monocytes in FIRES. Ann Clin Transl Neurol. 2023;10(5):719–31. 36924141 10.1002/acn3.51755 PMC10187718

[13] Kim ER, Kim SR, Cho W, Lee SG, Kim SH, Kim JH, et al. Short term isocaloric ketogenic diet modulates NLRP3 inflammasome via B-hydroxybutyrate and fibroblast growth factor 21. Front Immunol. 2022;13:843520. 35572519 10.3389/fimmu.2022.843520 PMC9095902

[14] Shang S, Wang L, Zhang Y, Lu H, Lu X. The beta-Hydroxybutyrate suppresses the migration of glioma cells by inhibition of NLRP3 inflammasome. Cell Mol Neurobiol. 2018;38:1479–89. 30218403 10.1007/s10571-018-0617-2 PMC11469911

[15] Goldberg EL, Shchukina I, Asher JL, Sidorov S, Artyomov MN, Dixit VD. Ketogenesis activates metabolically protective γδ T cells in visceral adipose tissue. Nat Metab. 2020;2(1):50–61. 32694683 10.1038/s42255-019-0160-6 PMC10150608

[16] Abdelhady R, Saber S, Ahmed Abdel-Reheim M, Mohammad S, Alamri M, Alfaifi J, et al. Unveiling the therapeutic potential of exogenous β-hydroxybutyrate for chronic colitis in rats. novel insights on autophagy, apoptosis, and pyroptosis. Front Pharmacol. 2023;14:1239025. 10.3389/fphar.2023.1239025 PMC10570820 37841914

[17] Wagner AS, Baumann SM, Semmlack S, Frei AI, Rüegg S, Hunziker S, et al. Comparing patients with isolated seizures and status epilepticus in intensive care units. Neurology. 2023;100:e1763–75. 36878696 10.1212/WNL.0000000000206838 PMC10136011

[18] Kishore AR, Michael TH. Inflammasomes in neurological disorders - mechanisms and therapeutic potential. Nat Rev Neurol. 2024;20(2):67–83. 38195712 10.1038/s41582-023-00915-x

[19] Alvim MKM, Morita-Sherman ME, Yasuda CL, Rocha NP, Vieira ÉL, Pimentel-Silva LR, et al. Inflammatory and neurotrophic factor plasma levels are related to epilepsy independently of etiology. Epilepsia. 2021;62(10):2385–94. 34331458 10.1111/epi.17023

[20] Vezzani A, Balosso S, Ravizza T. Neuroinflammatory pathways as treatment targets and biomarkers in epilepsy. Nat Rev Neurol. 2019;15(8):459–72. 31263255 10.1038/s41582-019-0217-x

[21] Zhou Z, Li K, Chu Y, Li C, Zhang T, Liu P, et al. ROS-removing nano-medicine for navigating inflammatory microenvironment to enhance anti-epileptic therapy. Acta Pharmaceutica Sinica B. 2023;13(3):1246–61. 36970212 10.1016/j.apsb.2022.09.019 PMC10031259

[22] Hanin A, Cespedes J, Dorgham K, Pulluru Y, Gopaul M, Gorochov G, et al. Cytokines in new-onset refractory status epilepticus predict outcomes. Ann Neurol. 2023;94(1):75–90. 36871188 10.1002/ana.26627

[23] DeSena AD, Do T, Schulert GS. Systemic autoinflammation with intractable epilepsy managed with interleukin-1 blockade. J Neuroinflammation. 2018;15(1):38. 29426321 10.1186/s12974-018-1063-2 PMC5807745

[24] Stredny CM, Case S, Sansevere AJ, Son M, Henderson L, Gorman MP. Interleukin-6 Blockade With Tocilizumab in Anakinra-Refractory Febrile Infection-Related Epilepsy Syndrome (FIRES). Child Neurol Open. 2020;7:2329048X20979253. 33403221 10.1177/2329048X20979253 PMC7745547

[25] Hsieh MY, Lin JJ, Hsia SH, Huang JL, Yeh KW, Chang KW, et al. Diminished toll-like receptor response in febrile infection-related epilepsy syndrome (FIRES). Biomed J. 2020;43(3):293–304. 32651134 10.1016/j.bj.2020.05.007 PMC7424096

[26] Ravichandran KA, Heneka MT. Inflammasomes in neurological disorders - mechanisms and therapeutic potential. Nat Rev Neurol. 2024;20(2):67–83. 38195712 10.1038/s41582-023-00915-x

[27] Tan CC, Zhang JG, Tan MS, Chen H, Meng DW, Jiang T, et al. NLRP1 inflammasome is activated in patients with medial temporal lobe epilepsy and contributes to neuronal pyroptosis in amygdala kindling-induced rat model. J Neuroinflammation. 2015;12:18. 25626361 10.1186/s12974-014-0233-0 PMC4314732

[28] Meng XF, Tan L, Tan MS, Jiang T, Tan CC, Li MM, et al. Inhibition of the NLRP3 inflammasome provides neuroprotection in rats following amygdala kindling-induced status epilepticus. J Neuroinflammation. 2014;11:212. 25516224 10.1186/s12974-014-0212-5 PMC4275944

[29] Wang X, Xiao A, Yang Y, Zhao Y, Wang CC, Wang Y, et al. DHA and EPA prevent seizure and depression-like behavior by inhibiting ferroptosis and neuroinflammation via diferent mode-of-actions in a pentylenetetrazole-induced kindling model in mice. Mol Nutr Food Res. 2022;66(22):e2200275. 36099650 10.1002/mnfr.202200275

[30] Xu X, Yin D, Ren H, Gao W, Li F, Sun D, et al. Selective NLRP3 inflammasome inhibitor reduces neuroinflammation and improves long-term neurological outcomes in a murine model of traumatic brain injury. Neurobiol Dis. 2018;117:15–27. 29859317 10.1016/j.nbd.2018.05.016

[31] Pohlentz MS, Müller P, Cases-Cunillera S, Opitz T, Surges R, Hamed M, et al. Characterization of NLRP3 pathway-related neuroinflammation in temporal lobe epilepsy. PLoS ONE. 2022;17(8):e0271995. 35972937 10.1371/journal.pone.0271995 PMC9380933

[32] Samadianzakaria A, Abdolmaleki Z, FASMmaleki F. The efect of valproic acid and furosemide on the regulation of the inflammasome complex (NLRP1 and NLRP3 mRNA) in the brain of epileptic animal model. Brain Res Bull. 2022;191:20–9. 36209957 10.1016/j.brainresbull.2022.10.002

[33] Qin Z, Song J, Lin A, Yang W, Zhang W, Zhong F, et al. GPR120 modulates epileptic seizure and neuroinflammation mediated by NLRP3 inflammasome. J Neuroinflammation. 2022;19(1):121. 35624482 10.1186/s12974-022-02482-2 PMC9137133

[34] Wang Z, Zhou L, An D, Xu W, Wu C, Sha S, et al. TRPV4-induced inflammatory response is involved in neuronal death in pilocarpine model of temporal lobe epilepsy in mice. Cell Death Dis. 2019;10(6):386. 31097691 10.1038/s41419-019-1612-3 PMC6522539

[35] Yue J, Wei YJ, Yang XL, Liu SY, Yang H, Zhang CQ. NLRP3 inflammasome and endoplasmic reticulum stress in the epileptogenic zone in temporal lobe epilepsy: molecular insights into their interdependence. Neuropathol Appl Neurobiol. 2020;46(7):770–85. 32311777 10.1111/nan.12621

[36] Gong L, Han Y, Chen R, Yang P, Zhang C. LncRNA ZNF883-mediated NLRP3 inflammasome activation and epilepsy development involve USP47 upregulation. Mol Neurobiol. 2022;59(8):5207–21. 35678979 10.1007/s12035-022-02902-7

[37] Yue J, He J, Wei Y, Shen K, Wu K, Yang X, et al. Decreased expression of Rev-Erbα in the epileptic foci of temporal lobe epilepsy and activation of Rev-Erbα have anti- inflammatory and neuroprotective efects in the pilocarpine model. J Neuroinflammation. 2020;17(1):43. 32005256 10.1186/s12974-020-1718-7 PMC6993411

[38] Cristina de Brito Toscano E, Leandro Marciano Vieira E, Boni Rocha Dias B, Vidigal Caliari M, Paula Gonçalves A, Varela Giannetti A, et al. NLRP3 and NLRP1 inflammasomes are up-regulated in patients with mesial temporal lobe epilepsy and may contribute to overexpression of caspase-1 and IL-β in sclerotic hippocampi. Brain Res. 2021;1752:147230. 33385378 10.1016/j.brainres.2020.147230

[39] Shen J, Tu L, Chen D, Tan T, Wang Y, Wang S. TRPV4 channels stimulate Ca2+ -induced Ca2+ release in mouse neurons and trigger endoplasmic reticulum stress after intracerebral hemorrhage. Brain Res Bull. 2019;146:143–52. 30508606 10.1016/j.brainresbull.2018.11.024

[40] Jimenez-Pacheco A, Diaz-Hernandez M, Arribas-Blázquez M, Sanz-Rodriguez A, Olivos-Oré LA, Artalejo AR, et al. Transient P2X7 receptor antagonism produces lasting reductions in spontaneous seizures and gliosis in experimental temporal lobe epilepsy. J Neurosci. 2016;36(22):5920. 27251615 10.1523/JNEUROSCI.4009-15.2016 PMC6601816

[41] Tracey D, Klareskog L, Sasso EH, Salfeld JG, Tak PP. Tumor necrosis factor antagonist mechanisms of action: a comprehensive review. Pharmacol Ther. 2008;117(2):244–79. 18155297 10.1016/j.pharmthera.2007.10.001

[42] Teixeira AL, de Souza RT, Zanetti MV, Brunoni AR, Busatto GF, Zarate CA Jr, et al. Increased plasma levels of soluble TNF receptors 1 and 2 in bipolar depression and impact of lithium treatment. Hum Psychopharmacol Clin Exp. 2015;30(1):52–6. 10.1002/hup.2450 PMC5858728 25572309

[43] Morin-Brureau M, Milior G, Royer J, Chali F, Le Duigou C, Savary E, et al. Microglial phenotypes in the human epileptic temporal lobe. Brain. 2018;141(12):3343–60. 30462183 10.1093/brain/awy276 PMC6277010

[44] Tröscher AR, Wimmer I, Quemada-Garrido L, Köck U, Gessl D, Verberk SGS, et al. Microglial nodules provide the environment for pathogenic T cells in human encephalitis. Acta Neuropathol. 2019;137(4):619–35. 30663001 10.1007/s00401-019-01958-5 PMC6426829

[45] Zhang S, Chen F, Zhai F, Liang S. Role of HMGB1/TLR4 and IL-1β/IL-1R1 Signaling Pathways in Epilepsy. Front Neurol. 2022;13:904225. 35837232 10.3389/fneur.2022.904225 PMC9274112

[46] Lin WS, Hsu TR. Hypothesis: Febrile infection-related epilepsy syndrome is a microglial NLRP3 inflammasome/IL-1 axis-driven autoinflammatory syndrome. Clin Transl Immunology. 2021;10(6):e1299. 34141434 10.1002/cti2.1299 PMC8204115

[47] Ulusoy C, Vanlı-Yavuz EN, Şanlı E, Timirci-Kahraman Ö, Yılmaz V, Bebek N, et al. Peripheral blood expression levels of inflammasome complex components in two diferent focal epilepsy syndromes. J Neuroimmunol. 2020;347:577343. 32731050 10.1016/j.jneuroim.2020.577343

[48] Song XJ, Han W, He R, Li TY, Xie LL, Cheng L, et al. Alterations of hippocampal myelin sheath and axon sprouting by status convulsion and regulating Lingo-1 expression with RNA interference in immature and adult rats. Neurochem Res. 2018;43(3):721–35. 29383653 10.1007/s11064-018-2474-2

[49] Rana A, Musto AE. The role of inflammation in the development of epilepsy. J Neuroinflammation. 2018;15(1):144. 29764485 10.1186/s12974-018-1192-7 PMC5952578

[50] Sivandzade F, Prasad S, Bhalerao A, Cucullo L. NRF2 and NF-қB interplay in cerebrovascular and neurodegenerative disorders: molecular mechanisms and possible therapeutic approaches. Redox Biol. 2019;21:101059. 10.1016/j.redox.2018.11.017 PMC6302038 30576920

[51] Xu T, Liu J, Li XR, Yu Y, Luo X, Zheng X, et al. The mTOR/NF-κB Pathway mediates neuroinflammation and synaptic plasticity in diabetic encephalopathy. Mol Neurobiol. 2021;58(8):3848–62. 33860440 10.1007/s12035-021-02390-1

[52] Zheng J, Lu J, Mei S, Wu H, Sun Z, Fang Y, et al. Ceria nanoparticles ameliorate white matter injury after intracerebral hemorrhage: microglia-astrocyte involvement in remyelination. J Neuroinflammation. 2021;18(1):43. 33588866 10.1186/s12974-021-02101-6 PMC7883579

[53] McDonald TJW, Cervenka MC. Ketogenic diets for adult neurological disorders. Neurotherapeutics. 2018;15(4):1018–31. 30225789 10.1007/s13311-018-0666-8 PMC6277302

[54] Koh S, Dupuis N, Auvin S. Ketogenic diet and neuroinflammation. Epilepsy Res. 2020;167:106454. 32987244 10.1016/j.eplepsyres.2020.106454

[55] Girardin ML, Flamand T, Roignot O, Abi Warde MT, Mutschler V, Voulleminot P, et al. Treatment of new onset refractory status epilepticus/febrile infection-related epilepsy syndrome with tocilizumab in a child and a young adult. Epilepsia. 2023;64(6):e87–92. 36961094 10.1111/epi.17591

[56] Jeong EA, Jeon BT, Shin HJ, Kim N, Lee DH, Kim HJ, et al. Ketogenic diet-induced peroxisome proliferator-activated receptor-γ activation decreases neuroinflammation in the mouse hippocampus after kainic acid-induced seizures. Exp Neurol. 2011;232(2):195–202. 21939657 10.1016/j.expneurol.2011.09.001

[57] Lu Y, Yang YY, Zhou MW, Liu N, Xing HY, Liu XX, et al. Ketogenic diet attenuates oxidative stress and inflammation after spinal cord injury by activating Nrf2 and suppressing the NF-kappaB signaling pathways. Neurosci Lett. 2018;683:13–8. 29894768 10.1016/j.neulet.2018.06.016

[58] Liśkiewicz AD, Liśkiewicz D, Marczak Ł, Przybyła M, Grabowska K, Student S, et al. Obesity-associated deterioration of the hippocampus is partially restored after weight loss. Brain Behav Immun. 2021;96:212–26. 34087424 10.1016/j.bbi.2021.05.030

[59] Ercan I, Cilaker Micili S, Soy S, Engur D, Tufekci KU, Kumral A, et al. Bilirubin induces microglial NLRP3 inflammasome activation in vitro and in vivo. Mol Cell Neurosci. 2023;125:103850. 36965549 10.1016/j.mcn.2023.103850

[60] Jayanti S, Dalla Verde C, Tiribelli C, Gazzin S. Inflammation, dopaminergic brain and bilirubin. Int J Mol Sci. 2023;24(14):11478. 37511235 10.3390/ijms241411478 PMC10380707

[61] Jayanti S, Moretti R, Tiribelli C, Gazzin S. Bilirubin prevents the TH+ dopaminergic neuron loss in a Parkinson’s disease model by acting on TNF-α. Int J Mol Sci. 2022;23(22):14276. 36430754 10.3390/ijms232214276 PMC9693357

[62] Sun W, Wang Q, Zhang R, Zhang N. Ketogenic diet attenuates neuroinflammation and induces conversion of M1 microglia to M2 in an EAE model of multiple sclerosis by regulating the NF-κB/NLRP3 pathway and inhibiting HDAC3 and P2X7R activation. Food Funct. 2023;14(15):7247–69. 37466915 10.1039/d3fo00122a

[63] Yin N, Zhao Y, Liu C, Yang Y, Wang ZH, Yu W, et al. Engineered nanoerythrocytes alleviate central nervous system inflammation by regulating the polarization of inflammatory microglia. Adv Mater. 2022;34(27):e2201322. 35483045 10.1002/adma.202201322

[64] Graff EC, Fang H, Wanders D, Judd RL. Anti-inflammatory effects of the hydroxycarboxylic acid receptor 2. Metabolism. 2016;65:102–13. 26773933 10.1016/j.metabol.2015.10.001

[65] Ghosh S, Castillo E, Frias ES, Swanson RA. Bioenergetic regulation of microglia. Glia. 2018;66(6):1200–12. 29219210 10.1002/glia.23271 PMC5903989

[66] Youm YH, Nguyen KY, Grant RW, Goldberg EL, Bodogai M, Kim D, et al. The ketone metabolite β-hydroxybutyrate blocks NLRP3 inflammasome-mediated inflammatory disease. Nat Med. 2015;21(3):263–9. 25686106 10.1038/nm.3804 PMC4352123

[67] Sukkar SG, Cogorno L, Pisciotta L, Pasta A, Vena A, Gradaschi R, et al. Clinical efficacy of eucaloric ketogenic nutrition in the COVID-19 cytokine storm: a retrospective analysis of mortality and intensive care unit admission. Nutrition. 2021;89:111236. 33895559 10.1016/j.nut.2021.111236 PMC7937042

[68] Sukkar SG, Bassetti M. Induction of ketosis as a potential therapeutic option to limit hyperglycemia and prevent cytokine storm in COVID-19. Nutrition. 2020;79–80:110967. 32942131 10.1016/j.nut.2020.110967 PMC7416786

[69] Bradshaw PC, Seeds WA, Miller AC, Mahajan VR, Curtis WM. COVID-19: Proposing a ketone-based metabolic therapy as a treatment to blunt the cytokine storm. Oxid Med Cell Longev. 2020;2020:6401341. 33014275 10.1155/2020/6401341 PMC7519203

[70] Dalbeth N, Gosling AL, Gaffo A, Abhishek A. Gout. Lancet. 2021;397(10287):1843–55. 33798500 10.1016/S0140-6736(21)00569-9

[71] Binobead MA, Aldakhilallah AH, Alsedairy SA, Al-Harbi LN, Al-Qahtani WH, Alshammari GM. Effect of low-carbohydrate diet on beta-hydroxybutyrate ketogenesis metabolic stimulation and regulation of NLRP3 ubiquitination in obese Saudi women. Nutrients. 2023;15(4):820. 36839178 10.3390/nu15040820 PMC9958539

[72] Kesarwani P, Kant S, Zhao Y, Miller CR, Chinnaiyan P. The Influence of the Ketogenic Diet on the Immune Tolerant Microenvironment in Glioblastoma. Cancers (Basel). 2022;14(22):5550. 36428642 10.3390/cancers14225550 PMC9688691

[73] Kesarwani P, Kant S, Zhao Y, Miller CR, Chinnaiyan P. Abstract 632: The immune consequences of a ketogenic diet in GBM and its therapeutic implications. Cancer Res. 2022;82(12_Supple):632–632.34921014

[74] Rahman M, Muhammad S, Khan MA, Chen H, Ridder DA, Müller-Fielitz H, et al. The beta-hydroxybutyrate receptor HCA2 activates a neuroprotective subset of macrophages. Nat Commun. 2014;5:3944. 24845831 10.1038/ncomms4944

[75] Murphy S. Abstract A57: Killing cancer with keto: Beta-hydroxybutyrate the main metabolite produced by a ketogenic diet acts as an endogenous histone deacetylase inhibitor to sensitize immunotherapy resistant prostate cancer to immune checkpoint blockade. Cancer Immunol Res. 2022;10(12 Supple):A57–A57.

[76] Pinto A, Bonucci A, Maggi E, Corsi M, Businaro R. Anti-oxidant and anti-inflammatory activity of ketogenic diet: new perspectives for neuroprotection in Alzheimer’s disease. Antioxidants. 2018;7(5):63. 29710809 10.3390/antiox7050063 PMC5981249

[77] Cheng CW, Biton M, Haber AL, Gunduz N, Eng G, Gaynor LT, et al. Ketone body signaling mediates intestinal stem cell homeostasis and adaptation to diet. Cell. 2019;178(5):1115-1131.e15. 31442404 10.1016/j.cell.2019.07.048 PMC6732196

[78] Huang C, Wang P, Xu X, Zhang Y, Gong Y, Hu W, et al. The ketone body metabolite β-hydroxybutyrate induces an antidepression-associated ramification of microglia via HDACs inhibition-triggered Akt-small RhoGTPase activation. Glia. 2018;66(2):256–78. 29058362 10.1002/glia.23241

[79] Qin L, Ma K, Yan Z. Rescue of histone hypoacetylation and social deficits by ketogenic diet in a Shank3 mouse model of autism. Neuropsychopharmacol. 2022;47(6):1271–9. 10.1038/s41386-021-01212-1 PMC9018860 34703011

[80] Kong G, Huang Z, Ji W, Wang X, Liu J, Wu X, et al. The ketone metabolite β-Hydroxybutyrate attenuates oxidative stress in spinal cord injury by suppression of class I histone deacetylases. J Neurotraum. 2017;34(18):2645–55. 10.1089/neu.2017.5192 28683591

[81] Shimazu T, Hirschey MD, Newman J, He W, Shirakawa K, Le Moan N, et al. Suppression of oxidative stress by beta-hydroxybutyrate, an endogenous histone deacetylase inhibitor. Science. 2013;339(6116):211–4. 23223453 10.1126/science.1227166 PMC3735349

[82] Shen Y, Kapfhamer D, Minnella AM, Kim JE, Won SJ, Chen Y, et al. Bioenergetic state regulates innate inflammatory responses through the transcriptional co-repressor CtBP. Nat Commun. 2017;8(1):624. 28935892 10.1038/s41467-017-00707-0 PMC5608947

[83] Saitoh M, Kobayashi K, Ohmori I, Tanaka Y, Tanaka K, Inoue T, et al. Cytokine-related and sodium channel polymorphism as candidate predisposing factors for childhood encephalopathy FIRES/AERRPS. J Neurol Sci. 2016;368:272–6. 27538648 10.1016/j.jns.2016.07.040

[84] Welzel T, Ziesenitz VC, Weber P, Datta AN, van den Anker JN, Gotta V. Drug-drug and drug-food interactions in an infant with early-onset SCN2A epilepsy treated with carbamazepine, phenytoin and a ketogenic diet. Br J Clin Pharmacol. 2021;87(3):1568–73. 32737897 10.1111/bcp.14503

[85] Kim DY, Simeone KA, Simeone TA, Pandya JD, Wilke JC, Ahn Y, et al. Ketone bodies mediate antiseizure effects through mitochondrial permeability transition. Ann Neurol. 2015;78(1):77–87. 25899847 10.1002/ana.24424 PMC4480159

[86] Chimienti G, Orlando A, Lezza AMS, D’Attoma B, Notarnicola M, Gigante I, et al. The ketogenic diet reduces the harmful effects of stress on gut mitochondrial biogenesis in a rat model of irritable bowel syndrome. Int J Mol Sci. 2021;22(7):3498. 33800646 10.3390/ijms22073498 PMC8037144

[87] Almekkawi AK, Sheets R, Werner B, Perryman R, Singh I, Karim M, et al. TMET-24 mitochondrial and gene expression changes in brain tumor cells grown in β-hydroxybutyrate. Neuro- Oncology. 2022;24(Supple7):vii266–7.

[88] Kong C, Yan X, Liu Y, Huang L, Zhu Y, He J, et al. Ketogenic diet alleviates colitis by reduction of colonic group 3 innate lymphoid cells through altering gut microbiome. Signal Transduct Target Ther. 2021;6(1):154. 33888680 10.1038/s41392-021-00549-9 PMC8062677

[89] Golonka RM, Xiao X, Abokor AA, Joe B, Vijay-Kumar M. Altered nutrient status reprograms host inflammation and metabolic health via gut microbiota. J Nutr Biochem. 2020;80:108360. 32163821 10.1016/j.jnutbio.2020.108360 PMC7242157

